# Space use of juvenile green turtles along the North Pacific coast of Costa Rica

**DOI:** 10.1371/journal.pone.0326473

**Published:** 2026-04-20

**Authors:** Fanqi Wu, Veronica Valverde-Cantillo, Chelsea Durr, Mario Espinoza, Maike Heidemeyer, Christopher G. Lowe, James R. Spotila, Frank V. Paladino

**Affiliations:** 1 Department of Biological Sciences, Purdue University Fort Wayne, Fort Wayne, Indiana, United States of America; 2 The Leatherback Trust, Fort Wayne, Indiana, United States of America; 3 Global Cause Foundation, Blacksburg, Virginia, United States of America; 4 Centro de Investigación en Ciencias del Mar y Limnología, Universidad de Costa Rica, San José, Costa Rica; 5 MigraMar, Bodega Bay, California, United States of America; 6 Equipo Tora Carey, El Jobo, La Cruz, Guanacaste, Costa Rica; 7 Department of Biological Sciences, California State University Long Beach, California, United States of America; 8 Department of Biodiversity, Earth and Environmental Science, Drexel University, Philadelphia, United States of America; Ocean Frontier Institute, CANADA

## Abstract

Understanding how threatened marine species use coastal areas and the extent of connectivity across different spatial and temporal scales is important for identifying critical habitats that can enhance conservation efforts in other regions of their distribution. Researchers in 2014 reported a previously unknown foraging ground for juvenile green turtles in the Gulf of Santa Elena, Costa Rica. However, there is limited information about this area and the turtles there. In this study, we investigated residency and space-use distribution of juvenile green turtles (*Chelonia mydas*) in the Gulf of Santa Elena, north Pacific coast of Costa Rica. We used acoustic telemetry to track 15 juvenile turtles (49–83 cm curved carapace length; CCL) for 19–628 days using 11 acoustic receivers placed within 5 habitat types: muddy areas, reef patches, macroalgae, rocky reefs, and mangroves. Residency Index varied among turtles, ranging from 0.17 to 1.00 (median = 0.72). It indicated a gradient from transient to high resident patterns during the study period. Space-use distribution with the Shannon index (H) ranged from 0.92 to 1.94 (median = 1.34). Large juvenile turtles exhibited significantly higher H than smaller turtles, indicating more even use of multiple stations, while small turtles showed more concentrated space use. However, the seasonal variation in H was not significant. The significant interaction between size and season suggested that there was a seasonal space-use pattern between large and small turtles.

## Introduction

The green turtle (*Chelonia mydas)* is currently classified as a least concern species globally [1], which is a big milestone in sea turtle conservation since it had been an endangered species for over 40 years since 1982. In the Pacific Ocean, the subpopulation of green turtle in the South Central Pacific is still classified as endangered, while subpopulations in the Southwest Pacific and the East Pacific are considered vulnerable [1]. The Pacific green turtle population is divided into two subgroups that can be recognized based on coloration: the yellow morphotype that breeds in the western Pacific (Yellow turtle) and the black morphotype that breeds in the eastern Pacific (Black turtle) [2–4]. The main morphometric differences between the two subgroups are the shape of the head, carapace, plastron, and flippers [4]. Both morphotypes spend their early pelagic stage in the open ocean until they grow to about 44 cm CCL, and then they settle in coastal foraging grounds as juveniles for their neritic stage [5].

During the pelagic stage, green turtles are primarily omnivorous. They feed on crustaceans, ctenophores, and jellyfish [6]. Once juvenile green turtles move into their coastal foraging grounds, they show high site fidelity to those areas. During this stage, juvenile green turtles show a shift in feeding behavior that entails a shift from an omnivorous diet to a predominantly herbivorous one, feeding on macroalgae, seagrass, and/or mangrove material [6–8]. When they become adults, they will find another foraging ground. The bays along the Pacific coast of Central America may be important developmental habitats for juvenile green turtles, and there is currently limited knowledge about these populations [9].

This study focused on determining the space use of juvenile yellow morphotype green turtles (hereafter juvenile green turtles) on the north Pacific coast of Costa Rica. Researchers in 2014 reported a previously unknown foraging ground for juvenile green turtles in the Gulf of Santa Elena, Costa Rica [10]. However, there is limited information about this area and the turtles there [10,11]. Our study was conducted in 2 bays in the Gulf of Santa Elena: Matapalito Bay and Santa Elena Bay. These two bays are connected to each other and provide a unique habitat combination of macroalgae, reef patches, rocky reefs, muddy areas, and mangroves. This area may be an important foraging ground for both morphotypes of green turtles, especially Matapalito Bay. Because of an ongoing conservation project with local fishermen sponsored by Equipo Toro Carey (ETC) and The Leatherback Trust (TLT), we had access to the study area and received invaluable assistance in data collection. Our study provided the first space-use data on juvenile green turtles in this region. These data provided important information that improves our understanding of the ecology of juvenile green turtles in this region and along the Pacific coast of Costa Rica. Our findings provided a basis for future sea turtle studies in this region and for conservation recommendations to protect green turtles in Northwest Costa Rica.

## Methods

### Study area

This study was conducted in Santa Elena Bay (SEB, 10°55’10.39“N, 85°47’52.88” W) and the adjacent Matapalito Bay (MPB, 10°55’58.75”N, 85°47’29.48”W) within the Gulf of Santa Elena, on the north Pacific coast of Costa Rica. This region experiences a tropical dry forest climate with strong seasonality driven by the Papagayo wind system [12,13]. The strong trade winds during the upwelling season (December-April) bring cold and nutrient-rich water into the two bays. As a result, shallow coastal waters have rich nutrients, low pH, and low oxygen levels, leading to a rapid growth of macroalgae [14,15]. During the non-upwelling season (May-November), the water temperature is warm and there are less macroalgae [16]. This area is protected from any overfishing or bycatch because it is in Santa Rosa National Park. Therefore, it makes an excellent place to study the ecology of green turtles in an undisturbed area.

The SEB is a semi-enclosed estuary located in the Gulf of Santa Elena. The bay has an area of 7.2 km^2^ and has an average depth of 15 m with a maximum depth of 40 m [17,18]. The habitats found in SEB mainly included mangrove along the coastal area of the inner Bay and rocky and coral reefs near the outer bay [19]. The MPB is adjacent to SEB, but it is much smaller, having an area of 0.72 km^2^. The habitats in MPB include rocky reefs near the entrance of the bay, macroalgae along the inner coastal shore, and reef patches in the western bay. We determined habitat types and areas by snorkeling surveys during each season. The habitats are 1.19 km^2^, and the rest of the bays with 6.73 km^2^ are open water areas.

### Acoustic receiver placement

**Fig 1:**
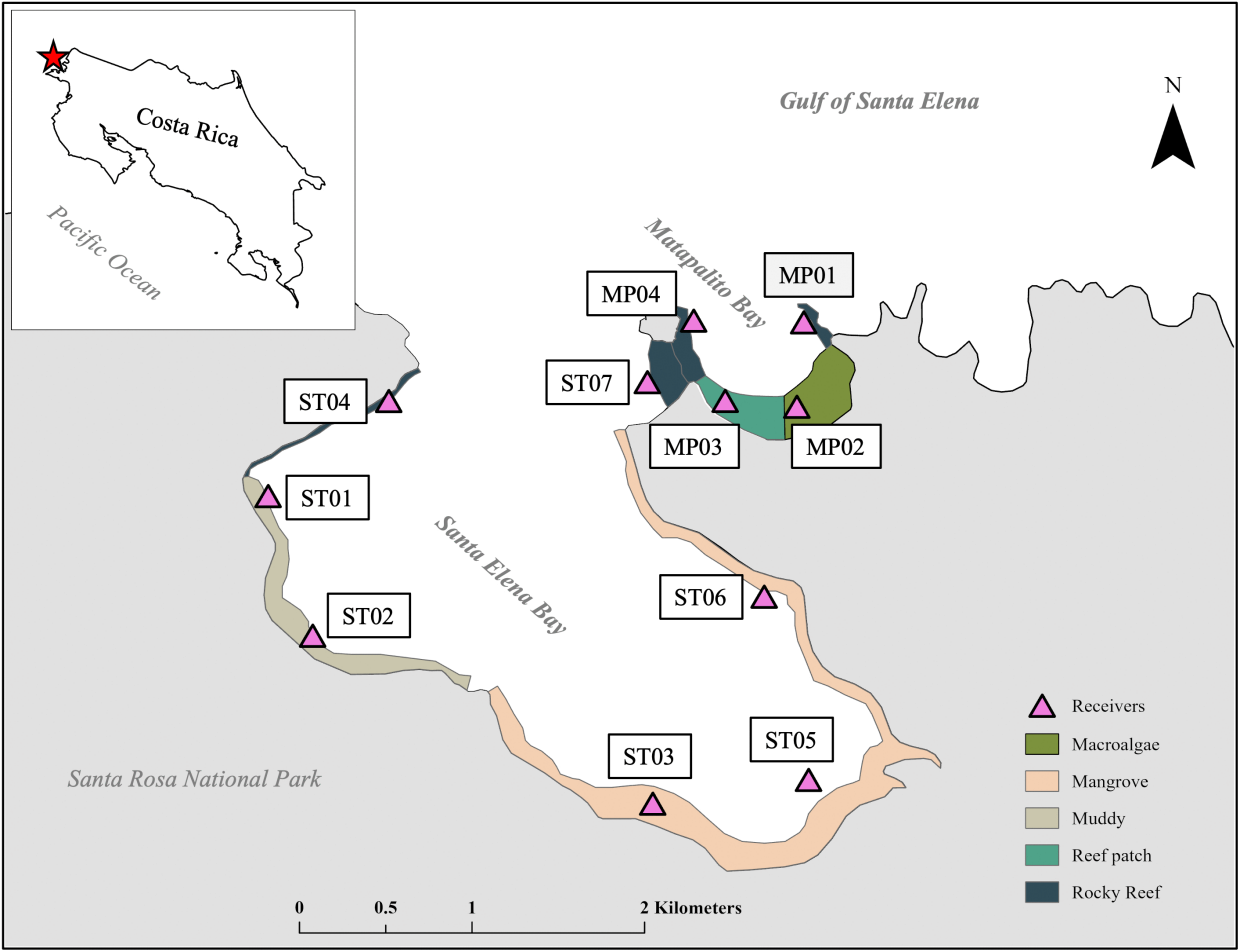
Receiver locations and habitats. Seven receivers were placed in Santa Elena Bay (SEB) and 4 receivers were placed in Matapalito Bay (MPB) along the north Pacific coast of Costa Rica. We digitized habitat areas and study area using ArcGIS Pro [20] and created the Costa Rica map with Natural Earth data (www.naturalearthdata.com).

We placed seven VR2Tx and four VR2W underwater receivers (InnovaSea Systems Inc., Nova Scotia, Canada) to record detections from turtles tagged with acoustic transmitters. In MPB, receiver (MP01 – MP04) distances ranged from 409 to 773 m with a mean nearest-neighbor distance of 454 m. The mean depth of the receivers was 6.2 m (2.9–9.6 m). MP01 and MP04 were placed at the eastern and western entrances of MPB, which were dominated by rocky reefs. MP02 was placed in the inner bay, which was dominated by macroalgae. MP03 was placed in the western part of the inner bay where it was dominated by reef patches. In SEB, receiver (ST01 – ST07) distance ranged from 854 to 3494 m, with a mean nearest-neighbor distance of 987 m. The mean depth of the receivers was 5.7 m (3.0–8.0 m). ST01 and ST02 were placed in the western SEB, where there were sandy and muddy areas. ST03, 05, and 06 were placed in the inner SEB, which was dominated by mangrove. ST04 was placed in the western SEB near the entrance of the Bay, while ST07 was placed in the eastern SEB near the entrance and covered the corridor between SEB and MPB (Fig 1). The detection range of the receivers was variable and influenced by the surrounding areas, with recorded ranges of less than 200 m in rocky and reef areas to up to 600 m in estuarine areas (V. Valverde unpublished data). Even though we placed receivers in different habitats, the detections only indicate that turtles were within the detection range of a receiver, but cannot confirm that turtles occupied a specific habitat related to that receiver. Detections may include deeper water areas or adjacent habitat. Given the estimated detection ranges and impacts of seasonal environmental changes, the coverage of nearshore habitat was partial and heterogeneous. Detection ranges may or may not overlap in some areas and do not provide continuous coverage across all areas, especially in Santa Elena Bay. Detection ranges may also extend beyond estimated habitat boundaries into adjacent deeper waters.

Using acoustic telemetry in movement studies and habitat studies is often limited to the detection probability, including locations and detection ranges. There is no standard method to filter out the false detections because transmitters are set up differently to meet study goals. The signal transmission is highly susceptible to interference from environmental factors, including wind, heavy rain, wave action, and biological noise [21,22]. For example, during the upwelling season, waves are larger than in the non-upwelling season; therefore, the detection range will be limited in the upwelling season. For fine-scale movement and habitat use studies, the number of receivers and coverage of study areas will be key drivers.

Each receiver recorded the date, time, and a unique ID code whenever it detected an animal tagged with a transmitter. Hourly water temperature was measured by the VR2Tx receivers (± 0.5 °C) or HOBO Water Temperature Pro v2 Data Loggers (Onset Computer Corporation; ± 0.2 °C) attached to the VR2W receivers. Prior to installation, we covered each receiver with a nylon stocking to prevent biofouling animals from attaching directly to the receiver. Receivers were supported on stainless steel bars or attached directly to a mooring line using cable ties and placed directly on the substrates. We manually downloaded data from the receivers every 2–3 months. Hereafter, we use “station” to represent the receivers.

### Turtle capture

We captured 15 yellow morphotype green turtles in Matapalito Bay in cooperation with field assistants from the ETC monitoring program. We used two methods to catch turtles: turtle tangle nets and by hand. Field assistants attached three nets together that totaled 240 m long and 8 m deep with a mesh size of 42 cm. The top of the net was held up with small buoys at 2 m intervals, and the bottom of the net was weighed down with small weights. If a turtle was caught in the nets, the net was light enough for the turtle to drag the whole net to the surface to breathe. We checked the nets every 30 min or less as well as when something appeared to be in the net. The nets were placed in the morning adjacent to the reef patch area near receiver MP03, where the water was about 11 m deep [10]. After placing the nets in the water, the ETC crew snorkeled in the shallow water areas nearby to look for turtles to hand capture.

Once caught, turtles were transferred to the research boat for morphometric data collection. First, we checked each turtle for metal identification tags (Style 681, National Band and Tag Company USFWS tags) and scanned the turtles with a PIT (Passive Integrated Transponder) tag scanner to see if the turtle had a PIT tag (FriendChip, Avid Identification Systems, Inc.). If it was a new turtle (i.e., did not have either a metal or PIT tag), we installed the tags and recorded each new animal. Next, we measured curved carapace length (CCL) and curved carapace width (CCW) using a soft measuring tape (± 0.1 cm). We measured CCL from the anterior point at the midline (nuchal scute) to the posterior tip of the supracaudal scute. We measured CCW across the widest part of the carapace. Finally, we measured the body mass of each turtle using a Rapaura 50 kg spring scale (± 0.1 kg) and a heavy-duty nylon rope.

### Acoustic transmitter installation

We installed VEMCO v16 coded acoustic transmitters (InnovaSea Systems Inc., Nova Scotia, Canada) on each turtle. Each transmitter had a length of 68 mm and a mass of 10.3 g in water. To reduce the detection collision probability, our transmitters sent a random signal pulse every 60–90 seconds. We installed each transmitter on a clean and flat area on the carapace just above the left or right rear flipper. We used a wire brush to clean the carapace and an alcohol pad to sanitize the area then used a 5/16” drill bit to drill 3 holes in the marginal scutes on the posterior carapace. We used stainless steel wire and cable crimp sleeves to tie the transmitter to the carapace and coated the base of the transmitter in marine glue. It took about an hour for the glue to dry, during which time the turtle was monitored on the boat. Once the transmitter was secured and the glue was dry, we released the turtle back into the water. When we saw the turtles in the water or recaptured them, there was no sign of infection from the procedure.

All procedures involving animals were reviewed and approved by the Institutional Animal Care and Use Committee of Purdue University (IACUC protocol number: 1804001737). The field work was conducted by a research team at the University of Costa Rica and Equipo Toro Carey that were approved by the Guanacaste Conservation Areas (ACG) of the Ministry of Environment of Costa Rica. No animals were sacrificed during this study. Turtles were handled for the minimum time necessary, and efforts were made to reduce stress, including covering the eyes to calm the turtles, keeping them moist, and immediate release after tagging.

### Data analysis

Acoustic detections were filtered by removing false detections before analysis. False detections were defined as isolated records not followed or preceded by additional detections of the same individual at the same receiver within 60 minutes [21,22]. Detection probability varies by distance from receivers, habitat characteristics, and environmental conditions. Therefore, to reduce potential bias, we analyzed our detection data based on detection days and proportional station use. Turtles were classified into 2 size classes, with ≥ 65 cm CCL for large turtles and < 65 cm CCL for small turtles [8]. The seasons were the upwelling season from December to April and the non-upwelling season from May to November. Because the detection ranges of adjacent receivers may overlap, a turtle could be detected at multiple stations within short time intervals. Therefore, we quantified detections recorded at different stations within short temporal windows. In addition, to reduce potential bias associated with overlap detections, we aggregated detections to daily presence at each station before calculating station use and proportional space-use. For each turtle, we calculated the monitoring duration (tagging day – last day of the study), tracking duration (first detection – last detection), the number of detected days, total detections, and average daily detections.

### Residency index (RI)

For each turtle, we calculated RI using the number of detection days divided by the individual monitoring period, defined as the number of days between the tagging date and the end of the study period [23]. The index ranges from 0 to 1, with higher values indicating greater residency within the study area. Periods without detection do not necessarily indicate turtles leaving the study area since they may be outside detection ranges or in areas with reduced detection probability.

### Space use

We calculated the proportional use of each station at the individual turtle level, dividing the number of detection days at a given station by the total number of detection days across all stations for an individual turtle, categorized by the size class. To look at the seasonal impact on space use, we applied the same approach for each season. Then, we used two metrics to describe individual space-use patterns: 1) number of stations used, defined as the number of stations at which an individual turtle was detected; 2) Shannon Index (H) of proportional use across all stations, using package *vegan* in R [24]. We also calculated the Shannon index (hereafter H_space) for each turtle across the entire monitoring period to describe overall space-use distribution. The seasonal Shannon index (H_season) was calculated for each individual within each season to evaluate temporal changes in space-use distribution. A higher H value indicated more even distribution of detections across stations, while a lower H indicated more concentrated use of fewer stations. In this study, we evaluated space use at the individual level.

We used a Wilcoxon rank-sum test to evaluate the differences in the size-based H (large vs. small). The effect sizes were calculated to quantify the magnitude of differences between the size groups. To test seasonal variation in H, we used a Wilcoxon signed-rank test that only included turtles detected in both seasons. The joint effects of size class and season on H were tested by a linear mixed model with H as the response variable. The size class, season, and their interaction were predictors in the model. Turtle ID was the random intercept. We used ANOVA Type III tests to assess the model’s significance.

### Habitat use

Each station was assigned to a category of dominant habitat. We calculated seasonal proportional habitat use by summing station proportional use within each habitat category for each turtle and then calculated the mean proportional use for each habitat that was stratified by size class and season. We did not conduct formal statistical tests because of the limitations of our receiver array. We only used these results to support the interpretation of space use patterns. We identified each habitat area by combining satellite images [25] from different seasons and field observations during snorkeling surveys and deploying receivers [26]. Then we digitized the area of each habitat type. Field observations were conducted using snorkeling transects in Matapalito Bay. We quantified vegetation for each season.

## Results

### Detection and residency index

We tracked 15 juvenile green turtles (CM01 – CM15) from 16 October 2020 to 3 September 2022. The CCL size of turtles ranged from 49 to 83 cm (Table 1). The large size class had 5 turtles with CCL ranging from 65 to 83 cm, while the small size class had 10 turtles with CCL ranging from 49 to 56 cm. Tracking duration of all turtles ranged from 19 to 628 days (median = 297 days). Detection days of all turtles ranged from 4 to 301 days (median = 162 days, Table 1). We removed 7,108 false detections, representing 1.8% of total detections. Because CM01 was only detected for 4 days in a 19-day tracking duration, we excluded it from our analyses. Therefore, only 14 turtles remained in our study. The tracking duration for small turtles (n = 9) was 51–628 days (median = 316), while for the large turtles (n = 5) it was 111–455 days (median = 128). The detection days for small turtles were 51–301 days (median = 205) and for large turtles were 77–187 days (median = 118).

**Table 1:** Turtle and Tracking Information.

Turtle ID	Size Class	CCL (cm)	First Detection	Last Detection	Tracking Duration (days)	Monitoring period (days)	Detection Days
CM01	small	56.0	10/17/20	11/4/20	19	688	4
CM02	small	55.0	12/6/20	8/25/22	628	637	159
CM03	small	53.5	12/6/20	7/30/22	602	637	207
CM04	large	65.0	1/27/21	11/19/21	297	585	187
CM05	large	66.0	3/16/21	6/13/22	455	537	162
CM06	large	83.0	6/16/21	10/4/21	111	445	77
CM07	small	53.3	8/21/21	6/13/22	297	379	166
CM08	small	49.8	10/12/21	9/3/22	327	328	295
CM09	small	53.3	10/12/21	8/23/22	316	328	205
CM10	small	54.3	3/16/21	8/8/22	511	328	301
CM11	small	64.9	10/12/21	8/17/22	310	328	268
CM12	small	49.0	3/26/22	9/3/22	162	162	161
CM13	large	71.5	4/29/22	9/3/22	128	129	118
CM14	large	65.0	4/29/22	9/3/22	128	129	115
CM15	small	50.5	7/15/22	9/3/22	51	51	51

15 juvenile turtles were captured in MPB, including 10 small turtles and 5 large turtles. Tracking duration was defined as the period from the first detection day to the last detection day of each turtle. CM01 only had 4 detection days in 19 tracking days.

The RI varied among turtles, ranging from 0.17 to 1.00 (median = 0.72). The RI for small turtles ranged from 0.25 to 1.00 (median = 0.82). The RI for large turtles ranged from 0.17 to 0.92 (median = 0.32). CM 15 exhibited the highest RI with 1.00, indicating continuous presence during its tracking period. CM 12, CM 10, and CM 13 all had RI above 0.9 (0.99, 0.92, and 0.92). CM 06 and CM 02 showed lower RI (0.17 and 0.25), indicating more transient use of the study area (Table 2).

**Table 2:** Residency index (RI) of juvenile green turtles by individual and size class.

Turtle ID	Size Class	RI
CM02	small	0.25
CM03	small	0.33
CM04	large	0.32
CM05	large	0.30
CM06	large	0.17
CM07	small	0.44
CM08	small	0.90
CM09	small	0.63
CM10	small	0.92
CM11	small	0.82
CM12	small	0.99
CM13	large	0.92
CM14	large	0.89
CM15	small	1.00

The residency index was calculated as the proportion of days each turtle was detected relative to its monitoring period. RI ranges from 0 to 1, where higher values indicate more consistent presence within the study area and lower values indicate more intermittent use.

### Space use

**Fig 2:**
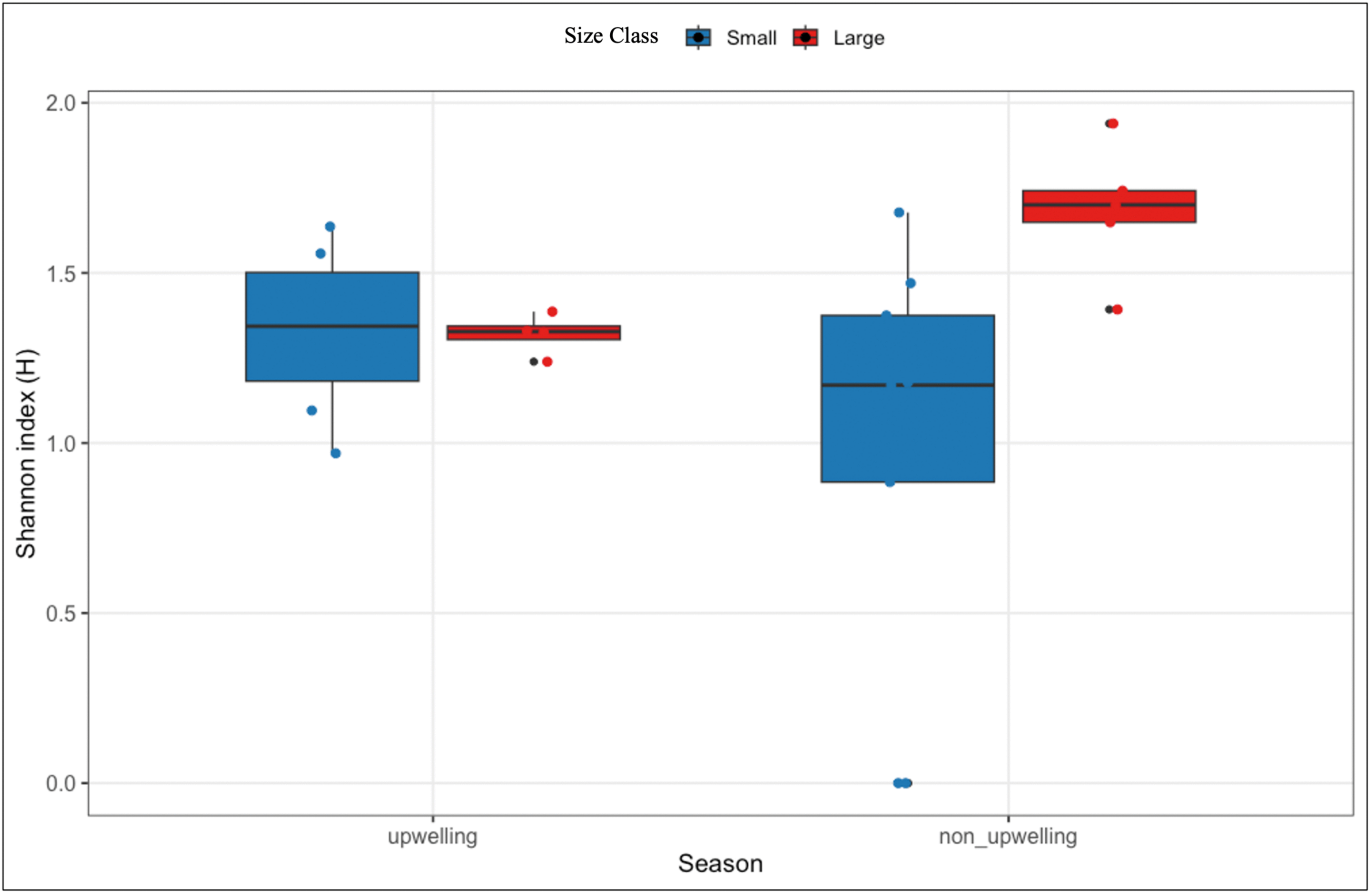
Shannon index (H) space use distribution between size classes across seasons: Shannon index (H) of space-use distribution in juvenile green turtles across size classes (large turtle in red color and small turtle in blue color) and seasons. Higher H values indicate a more even distribution of detections across receiver stations, whereas lower values indicate detections concentrated at fewer stations.

Space use also varied among turtles, with detections ranging from 4 to 11 stations. The number of detection days per station ranged from 1 to 270. The Shannon index of space use (H_space_) described the distribution of detections across all stations for each turtle. H_space_ for all turtles ranged from 0.92 to 1.94 (median = 1.34). Small turtles showed lower H_space_ (0.92–1.67, median = 1.27) compared to large turtles (1.34–1.94, median = 1.67). This difference was tested by the Wilcoxon rank-sum test (W = 4, p = 0.012, r = 0.66). The Shannon index of seasonal distribution of detections (H_season_) only for turtles detected in both seasons ranged from 0 to 1.74 (median = 1.33). CM02 and CM03 exhibited H_season_ with zero in non-upwelling, indicating they were detected by only one station during that season. There was no significant difference between seasons from the Wilcoxon signed-rank test (V = 30, p = 0.52, r = 0.20). The linear mixed model showed no significant difference between H and size class (p = 0.97) or seasons (p = 0.076). However, the interaction between size class and season was significant (p = 0.036), which means that seasonal changes in the distribution of space use differed between small and large turtles (Fig 2).

### Habitat use

**Fig 3:**
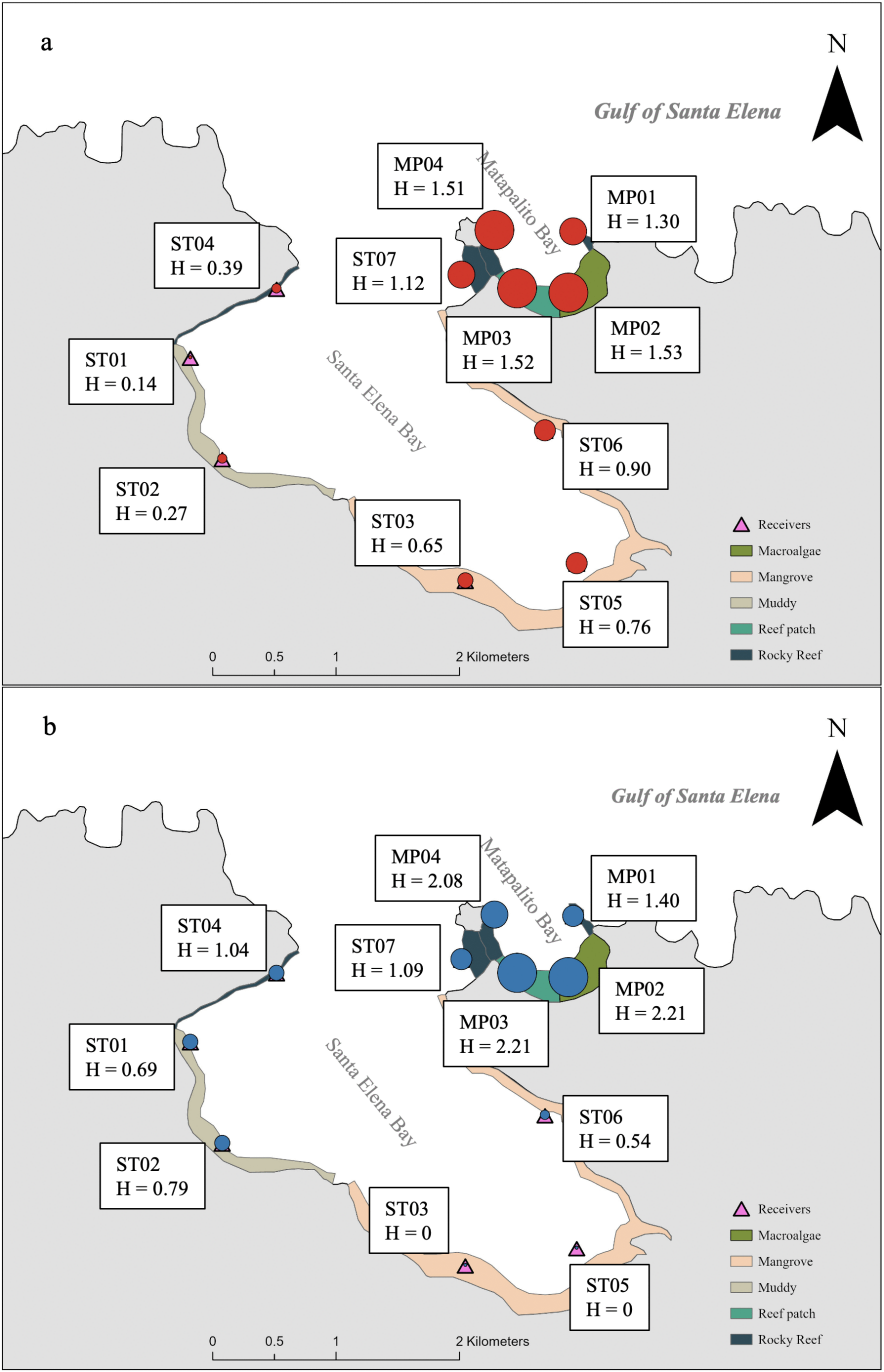
Spatial variation in Shannon index (H) of station use by juvenile green turtles across size classes. H values were calculated for each station based on the distribution of detections among individuals within large (3a) and small (3b) size classes. Higher H values indicate that multiple individuals used a station more evenly, whereas lower values indicate that station use was dominated by fewer individuals. An H value of 0 indicates that one turtle contributed detections at that station. The size of the circle is a relative indication of the H value.

Small turtles were detected across a broader range of stations during the upwelling season. During the non-upwelling season, CM02 and CM03 were detected exclusively at stations associated with muddy areas. During the upwelling season, the distribution of large turtle detection was among stations associated with rocky reef (Shannon index median = 0.40), macroalgae (median = 0.30), and reef patch (median = 0.29). During the non-upwelling season, their detection exhibited a wider distribution across stations associated with rock reef (median = 0.36), macroalgae (median = 0.22), reef patch (median = 0.14), mangrove (median = 0.18), and sandy area (median = 0.02). Overall, both size classes showed even distribution of detections at MP02 (H median for small turtle = 2.21 and for large turtles = 1.53) and MP03 (median for small turtle = 2.21 and for large turtles = 1.52) stations, which were associated with macroalgae and reef patch areas. Stations associated with mangrove contributed a small proportion of detections in both size classes (Fig 3).

Sea water temperatures ranged from 15.0 to 40.0 °C. Monthly mean water temperatures ranged from 20.3 to 29.2 °C (mean ± SD: 26.5 ± 2.2 °C) during the study period. During the upwelling season, strong winds blowing offshore (Papagayo winds) brought cold water into the bays. The monthly mean water temperature was from 21.4 °C to 25.6 °C (23.8 ± 1.4 °C) in MPB and from 20.3 to 27.4 °C (24.6 ± 1.8 °C) in SEB. During the non-upwelling season, the monthly mean water temperature increased from 26.3 to 29.2 °C (27.8 ± 0.7 °C) in MPB and from 26.1 to 29.2 °C (28.1 ± 0.7 °C) in SEB.

## Discussion

Detections of juvenile green turtles varied in both monitoring duration and detection frequency among individuals. Small turtles were detected over longer periods and on more days than the large turtles. The high detection rates observed in CM04 indicated strong use of specific areas within the receiver array. CM06, the largest juvenile (CCL = 83.0 cm), was in the study area for 77 days during the first 100 days of its tracking period. Overall, these results highlight individual variability in space use and suggest potential size-related differences in how they used the study area.

### Residency index

The residency index results were consistent with the pattern observed in the detection histories of each turtle. CM15, CM12, and CM10 had the highest RI values, indicating persistent use of the study area. In contrast, CM06 and CM02 had the lowest RI values. These results indicated heterogeneity in juvenile turtles using the study area, ranging from highly resident individuals to those that were periodically present. Our small turtles, except CM02, CM03, and CM07, showed similar RIs with juvenile green turtles reported in Port Curtis, Australia [26,27]. A higher value in RI suggested that those turtles consistently used the monitored area, while a low RI value indicated less frequent use of the area or broader movement beyond receiver coverage. Because both animal movement and the detection ability of receivers can influence acoustic detection, these findings likely reflect a combination of behavioral variation and detection probability. However, both detection and RI results in this study suggested that juvenile green turtles exhibit continuous space-use strategies, which also indicated that they may also use other bays in the Gulf as foraging grounds. Because receivers did not fully cover the study area and detection probability varied across spaces and seasons, we did not interpret the no detection period as definitive absence from this study area.

### Space use

The distribution of space use showed individual variation in how turtles used the study area. Differences in space-use distribution between size classes indicated that larger turtles used the study area more evenly across stations, whereas smaller turtles tended to concentrate their use within a limited number of locations. This pattern suggests a shift from spatially restricted to more distributed space use with increasing body size. Importantly, a significant interaction between size class and seasons indicated that small turtles exhibited greater variability in space use distribution between upwelling and non-upwelling seasons, especially highly concentrated use during the non-upwelling season. The large turtles maintained a consistent space use distribution across seasons. The lower H in large turtles in the upwelling season indicated that their space use was concentrated in fewer stations than in the non-upwelling season. This could be driven by food abundance. Therefore, we conclude that small turtles are more responsive to seasonal variations while larger turtles show more stable patterns of space use within the study area. Combined with the results of station use of large turtles, we found that large turtles had a large proportional use in areas of MP02 – MP04 in both seasons.

Similar findings have been reported in other studies in the Atlantic Ocean [28–30] and Indian Ocean [31,32]. Griffin et al. reported that their large juvenile green turtles left their normal activity areas on brief trips [33]. CM06, the largest juvenile (CCL = 83 cm), was in the study area for 77 days during the first 100 days of its tracking period. After that, it rarely visited the study area. CM02, CM03, and CM05 behaved in a similar fashion. This suggests that some individuals may have other activity areas outside MPB and SEB. Overall, our space use results together with detection results revealed that juvenile green turtles may use other bays in the Gulf of Santa Elena.

### Habitat use

Detection patterns based on station-associated habitats varied between size classes and across seasons and suggested differences in how size classes distributed their space use within the receiver array. The detections indicated the presence within detection ranges of receivers rather than habitat occupancy. Therefore, our results reflect relative space use in relation to receiver locations rather than habitat selection.

CM02 and CM03 during the non-upwelling season were primarily detected in the range of stations associated with muddy habitats, especially ST02. This is similar to turtles in in St Joseph Bay in Florida, USA, where juvenile green turtles were found in seagrass beds and open sandy areas [30]. Seasonal differences in detection patterns may reflect shifts in movement or distribution in the study area. During the upwelling season, small turtles were detected across a greater number of receivers, while detections of large turtles were more frequently recorded by receivers in macroalgae, reef patch, and rocky reef habitats. Our detection results matched the observations on juvenile green turtles in Coconut Island, USA and Sanriku Coast, Japan, which primarily feed on macroalgae, reef patches, and coral-covered areas [34–36]. One of the reasons that could explain this is intra-specific competition if food becomes limited [37,38], since large juvenile turtles are in diet transition to adult, consuming more vegetation [39,40]. However, our findings suggest that those juvenile green turtles were barely detected in the mangrove area, which is different from those in Australia [5].

## Further study and conservation recommendations

To our knowledge, our study provided the first description of the yellow morphotype juvenile green turtle space use along the Pacific coast of Central America; however, there are still a lot of remaining questions, especially on the habitat use of juvenile green turtles and interspecies interactions in the Gulf of Santa Elena. In this study area, we also found hawksbill sea turtles and black morphotype green turtles that only spend their life in the East Pacific. First of all, we recommend that future studies on sea turtles in this area should include a broader spatial scale, conduct a habitat survey, and develop fine-scale habitat use studies. The Gulf of Santa Elena includes multiple bays, and they are connected by open water. Based on our observation, we noticed that other bays also provide a certain level of food availability. For example, Leoncillios Bay connected to MPB by open water also has macroalgae and rock reef habitats. Even though this bay is much smaller than MPB, it could be a potential habitat for juvenile green turtles. Clyde-Brockway et al. (2022) also observed turtles in this area following fishing boats and consuming the fish from the boats [11].

Our results revealed the success of Costa Rica’s marine ecology protection and management program. The study area is under the management of the Santa Rosa National Park, with restrictions on fishing and water activities. However, there are more bays like MPB and SEB that are suitable feeding grounds for juvenile green turtles that are not under protection along the northwest of Costa Rica and Central America. Further study is needed to determine the use of these bays by sea turtles.
